# Resource-Efficient Design and Implementation of Real-Time Parking Monitoring System with Edge Device

**DOI:** 10.3390/s25072181

**Published:** 2025-03-29

**Authors:** Jungyoon Kim, Incheol Jeong, Jungil Jung, Jinsoo Cho

**Affiliations:** 1 Department of IT Convergence Engineering, Gachon University, Seongnam 13120, Republic of Korea; ekg1229@gachon.ac.kr (J.K.); kaddo@gachon.ac.kr (I.J.); 2 PCT Co., Ltd., Seongnam 13449, Republic of Korea; ceo@powerct.kr; 3 Department of Computer Engineering, Gachon University, Seongnam 13120, Republic of Korea

**Keywords:** dual-trigger system, edge device, smart parking system, vehicle occupancy detection, image processing

## Abstract

Parking management systems play a crucial role in addressing parking shortages and operational challenges; however, high initial costs and infrastructure requirements often hinder their implementation. Edge computing offers a promising solution by reducing latency and network traffic, thus optimizing operational costs. Nonetheless, the limited computational resources of edge devices remain a significant challenge. This study developed a real-time vehicle occupancy detection system utilizing SSD-MobileNetv2 on edge devices to process video streams from multiple IP cameras. The system incorporates a dual-trigger mechanism, combining periodic triggers and parking space mask triggers, to optimize computational efficiency and resource usage while maintaining high accuracy and reliability. Experimental results demonstrated that the parking space mask trigger significantly reduced unnecessary AI model executions compared to periodic triggers, while the dual-trigger mechanism ensured consistent updates even under unstable network conditions. The SSD-MobileNetv2 model achieved a frame processing time of 0.32 s and maintained robust detection performance with an F1-score of 0.9848 during a four-month field validation. These findings validate the suitability of the system for real-time parking management in resource-constrained environments. Thus, the proposed smart parking system offers an economical, viable, and practical solution that can significantly contribute to developing smart cities.

## 1. Introduction

Urbanization and the increase in the number of vehicles have caused a shortage of parking spaces, making parking management issues a significant societal challenge worldwide. According to the automotive industry research firm Hedges & Company, the number of cars has reached approximately 1.47 billion globally, with an annual growth rate of 4% [1], resulting in a severe shortage of parking spaces. Towne Park, a North American parking management provider, has reported that an average American commuter spends 17 h annually searching for parking spaces, costing USD 345 per driver in wasted time, fuel, and emissions [2].

To solve these problems, parking monitoring systems have been proposed to monitor the availability of parking spaces and provide drivers with information on empty parking spaces, thereby reducing parking time and alleviating traffic congestion. However, existing systems have high initial installation costs and require technical infrastructure, such as installing sensors and cameras and building data processing servers, as well as many limitations, especially when applied to existing parking lots. In addition to high initial costs, limitations in terms of scalability and maintenance have been noted.

Edge computing has emerged as an effective technology for overcoming these challenges. By processing data locally, edge computing reduces latency and network traffic, thereby offering cost savings and the benefits of real-time processing [3]. For instance, the processing of data on edge devices can reduce the delays and traffic that occur during transmission to the cloud. However, due to the limited computing resources of edge devices, efficient resource utilization is crucial. Research conducted by the Association for Computing Machinery (ACM) highlights that the limited resources of edge devices remain a challenge [4].

This study focused on designing and implementing a real-time parking monitoring system that can be quickly realized at low cost, focusing on improving the reliability and efficiency of parking occupancy in resource-constrained environments. To this end, this study developed an efficient framework that combines low-cost infrastructure, low computational requirements, real-time performance, and a state-sensitive trigger algorithm. The system seeks to overcome the limitations of existing systems, while efficiently utilizing the limited resources of edge devices, and providing economical and reliable real-time information to end users. The main contributions of this study are as follows:

1. Modular design of the proposed system: The proposed system entails three general modules that are compatible with all AI-based architectures rather than relying on specific use cases. The first module, the IP camera frame capture thread, implements stable and reliable image quality management and data capture through FFmpeg-based data processing, ensuring real-time performance. The second module determines whether a vehicle is occupied by a parking space by using an AI model. It can simultaneously determine the status of multiple parking spaces with a single image and is compatible with various AI models so as to ensure flexibility and scalability. The last module transmits data to the central server only when there exists a difference between the previous state of the parking space and its recognized current state, thereby reducing the network load and minimizing transmission costs.
2. Efficient data processing and reduced computational load: We devised a novel dual-trigger algorithm that combines the parking lot mask trigger and periodic trigger to efficiently use computing resources. Through the parking lot mask trigger, parking lot status changes can be sensitively detected in real-time, thereby reducing the frequency of AI model calls and effectively reducing the computational load on edge devices.
3. Field verification and performance evaluation: The proposed system was operated for 4 months in an actual parking lot with a total of 12 parking spaces, exhibiting stable performance in various scenarios.
4. Economical and scalable solution: The aforementioned contributions have significant implications in real-world parking management scenarios, demonstrating the economic effectiveness and practicality of the system. The proposed system, built using only edge devices and IP cameras, has remarkably reduced initial installation costs and can be deployed faster than traditional sensor-based solutions, demonstrating real scalability for smart city implementations.

The rest of the paper is organized as follows. Section 2 reviews the related studies, highlighting their methodologies and limitations. Section 3 presents the proposed system architecture, including the trigger algorithms and parking decision module. Section 4 describes the experimental setup, detailing the edge device, testing environment, and configuration. Section 5 provides an in-depth evaluation and discussion of the system’s performance, covering trigger algorithms, edge model efficiency, and real-world validation. Finally, Section 6 concludes this study with key findings and potential future improvements.

## 2. Related Research

### 2.1. Parking Occupancy Detection

Several studies have been conducted to guide parking occupancy. Table 1 compares different research papers on vehicle occupancy detection, including their respective objectives, the datasets used, the approaches employed, and the results obtained. The research on parking occupancy technology can be broadly divided into sensor-based solutions [5,6,7,8,9,10,11,12,13,14] and vision-based solutions [15,16,17,18,19,20,21,22,23].

In sensor-based solutions, the occupancy status is detected by placing sensor nodes in each parking space. Magnetic, ultrasonic, and infrared are the typically used sensors. However, these solutions require multiple sensors to detect several parking spaces, with a sensor node deployed per parking space. Moreover, small, robust, low-power, and cost-effective sensors must be used. Yang et al. [5] proposed a flexible magnetoelectric sensor that integrates Ecoflex elastomers with conductive structures, enabling a self-powered sensor system for parking space detection. Their study demonstrated improved output performance and response time, making these sensors a viable alternative for real-time parking occupancy monitoring. Allbadi et al. [6] developed a smart parking system utilizing ultrasonic and infrared sensors, controlled by an Arduino Mega 2560. Their system incorporated an RFID-based entry authorization mechanism and a WiFi-enabled mobile application, allowing users to check for available parking spaces remotely. The implementation, tested on a small-scale model, successfully simulated real-world parking scenarios by integrating sensor data with an LCD display and a mobile application interface.

Recently, parking occupancy detection technology using LiDAR sensors has been introduced, and its accuracy and reliability have been further improved. LiDAR sensors provide high-resolution distance and depth data, ensuring high reliability even in complex environments. Li et al. [14] proposed a system that accurately detects the real-time occupancy status of parking spaces using 3D LiDAR data and demonstrated its advantage of being robust to environmental noise.

These studies highlight the potential of sensor-based parking solutions, offering high precision and automation capabilities. Sensor-based detection algorithms mostly operate as pipelines that use a threshold calculation method of the signal as input. This method has played an important role in determining parking occupancy but has shown limited usability in large-scale systems due to the physical limitations of sensor technology and the burden of installation costs. To overcome these limitations, vision-based solutions have been explored as a cost-effective and scalable alternative, allowing a single camera to monitor multiple parking spaces while eliminating the need for per-space sensor deployment.

Vision-based solutions are proposed to overcome the limitations of sensor-based solutions by enabling the detection of multiple parking spaces with a single camera, reducing installation costs, and making them more useful in complex parking management scenarios. Image and video data obtained through cameras can provide additional information beyond parking space detection, thus enabling more precise parking management. Furthermore, the system does not require parking lot closures or the installation of extensive physical infrastructure, providing considerable flexibility. De Almeida et al. [15] proposed a system that effectively classifies parking spaces using the PKLot dataset. They employed texture techniques, such as local binary pattern (LBP) and local phase quantization (LPQ), to detect the presence of vehicles in parking spaces, with an accuracy rate of 89%. Baroffio et al. [16] utilized hue histograms and support vector machines (SVMs) to achieve real-time processing and high accuracy on validation data. Bulan et al. [17] designed a pipeline based on background subtraction and an SVM, known for its high performance and robustness to occlusion. However, these methods may exhibit unstable performance in complex environments.

Since the introduction of deep learning, researchers have been improving parking detection performance using models such as convolutional neural networks (CNNs) [18,19,20]. For instance, Acharya et al. [20] identified parking space occupancy using features extracted from pre-trained deep CNNs and successfully detected parking space status with an SVM classifier. Xie et al. [21] designed a system to detect the status of parking spaces in real-time using an improved YOLO algorithm, and Padilla Carrasco et al. [22] effectively detected small vehicles by combining T-YOLO and a multi-scale CNN.

However, using this method frequently generates a substantial volume of data, leading to increased data transmission costs and potential reliability issues. To address this issue, edge computing technology has been introduced, which processes data on local devices before transmitting it to a central server, thereby reducing network load and increasing processing speed. Vitek and Melničuk [23] implemented a histogram of gradients (HOG)-based classifier on Internet of Things (IoT) devices; however, manually crafted HOG features could result in significant errors during actual parking. Ke et al. [24] optimized data transmission by sending detected results from the edge to the server for matching and status determination, thereby reducing the computational load on the edge devices. In addition, Falaschetti et al. [25] optimized vehicle and pedestrian detection systems for embedded environments using the compressed Tiny YOLO v3 architecture and simultaneously secured real-time and accuracy. Ming et al. [26] proposed a method to detect street parking space occupancy using image recognition based on YOLO and demonstrated high accuracy in various environments. These studies suggest the economic feasibility and scalability of AI-based parking management systems and show the possibility of operating in large-scale environments at low cost.

**Table 1:** Literature survey on vision-based method.

Reference	Region	Purpose	Dataset	Method	Results
[15]	Brazil	Detecting parking occupancy using texture descriptor (local phase quantization, LPQ) for parking occupancy detection	RGB images (Data: 695,899 images captured from digital camera; PKLot)	Local binary pattern (LBP) and local phase quantization	Accuracy of 89%
[16]	Italy	Distributed parking lot occupancy detection using color histograms and linear support vector machines (SVMs)	Normalized hue histograms extracted from labeled data	Hue histogram, binary linear SVM classifier	Accuracy of 87%
[17]	USA	Efficient video processing algorithm-based vehicle occupancy detection for video-based real-time parking occupancy detection system	RGB images (Data: 1800 frames captured from a digital camera in various environmental conditions)	Background estimation and subtraction, motion detection, occlusion detection, and localization	Accuracy of 93.9%
[18]	Europe	Use a trained CNN for each parking space to determine occupancy status	RGB images (Data: PKLot, 12,584 images captured from a digital camera; CNRPark)	CNN (mAlexNet and mLeNet)	Accuracy of 89.9%
[19]	Indonesia	Real-time parking occupancy detection using only IP cameras using multiple deep learning architectures	RGB images (Data: CNRPark-Ext)	CNN (LeNet, AlexNet, mLeNet, and mAlexNet)	Accuracy of 93.15%
[20]	Australia	Identify parking space occupancy using features extracted from pre-trained deep CNN and detect parking occupancy using the SVM classifier	RGB images (Data: PKLot, 24,300 images captured from DSLR camera; Barry Street)	Pre-trained deep CNN and SVM	Accuracy of 96.7%
[23]	Czech Republic	Distributed a wireless camera system for determining parking space occupancy based on information from multiple cameras	RGB images (Data: about 1000 images collected in different daytimes and weather conditions)	Histogram of gradient (HOG)-based classifier and SVM	Accuracy of 91%
[24]	China	Using edge computing for parking occupancy detection using real-time video feeds	RGB images (Data: Pascal VOC, MIO-TCD)	SSD, background (BG) modeling detector, SORT, and fusion	Accuracy of 95.6%
[27]	Pakistan	Predicting parking locations using the deep extreme learning machine (DELM) approach to determine appropriate parking zones for drivers	RGB images (Data: 34,718 images from the PKLot)	DELM	Accuracy of 91.25%
[28]	Sweden	Real-time vehicle occupancy measurement using thermal imaging cameras and deep learning technology	RGB images (Data: 600 frames captured using a thermal camera based on motion detection in various environmental conditions)	YOLOv2, YOLO-Conv, GoogleNet, ResNet18, and ResNet50	Accuracy of 96.16%

This paper proposes a novel approach to overcome the major limitations of existing vision-based systems: high computational cost, network load due to data transmission, and low stability in complex environments. The proposed system adopts a modular design that is compatible with all AI architectures, providing an economical and scalable parking management solution. In particular, it is designed to operate efficiently even in limited resource environments, minimize the frequency of AI model calls based on a dual-trigger algorithm, reduce computational load, and reliably detect parking occupancy status in real-time.

### 2.2. Edge Device-Based Object Detection

Edge device-based object detection technology focuses on providing high accuracy and real-time performance while utilizing limited computing resources. It plays a key role in various application areas such as traffic control, safety detection, and smart city infrastructure construction. In particular, real-time object detection requires efficient data processing, and minimizing network delay and power consumption is essential.

The field of deep learning-based object detection has made significant progress with the advent of the R-CNN (regions with CNN features) model in 2013. Although the R-CNN significantly improves object detection performance compared to previous models, its processing speed is too slow for real-time applications. The processing times for a single image were found to be approximately 13 s and 53 s in GPU and CPU environments, respectively [29]. Subsequently, researchers have developed various deep learning-based models to enhance processing speeds.

**Figure 1:**
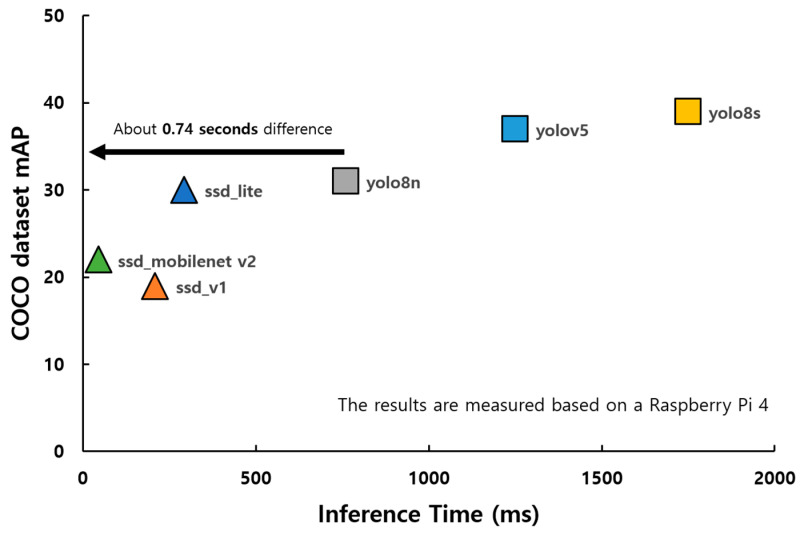
Performance comparison between SSD and YOLO algorithms in Raspberry Pi 4 [33,34,35].

Object detection models are broadly categorized into one-stage and two-stage models. Two-stage models, such as the faster R-CNN [30], initially generate candidate spaces and subsequently classify the objects within those spaces. While this approach provides high accuracy, it has significant computational demands, thereby making real-time processing challenging. This is especially problematic in resource-constrained environments such as edge devices. By contrast, one-stage models reduce the computational burden by simultaneously predicting object locations and classes without generating candidate spaces. Notable examples include YOLO [31] and SSD [32], which have adopted this structure. The performance of these algorithms is visually represented in Figure 1.

Recent studies tend to prefer the one-stage model considering the real-time performance of edge devices. Ming et al. [26] proposed a method to detect occupancy of street parking spaces using YOLOv8-based object detection and achieved high mean average precision (mAP). Falaschetti et al. [25] demonstrated the real-time processing capability of edge devices by implementing an object detection system optimized for embedded environments by compressing Tiny YOLO v3. These studies demonstrate the advantages of the one-stage model in applications where real-time performance is important.

In this study, we conducted a performance comparison experiment in a limited resource environment targeting YOLOv8n, YOLOv8n’s ONNX conversion and FP16 optimization version, and the SSD-MobileNet v2 model. By evaluating the inference speed, inference time, accuracy, and resource usage of each model, we selected the optimal model that can provide real-time performance and high accuracy at the same time even in a limited environment. Thus, we verified the possibility of implementing an efficient and economical object detection system in a limited environment such as an edge device.

## 3. Proposed System Design

**Figure 2:**
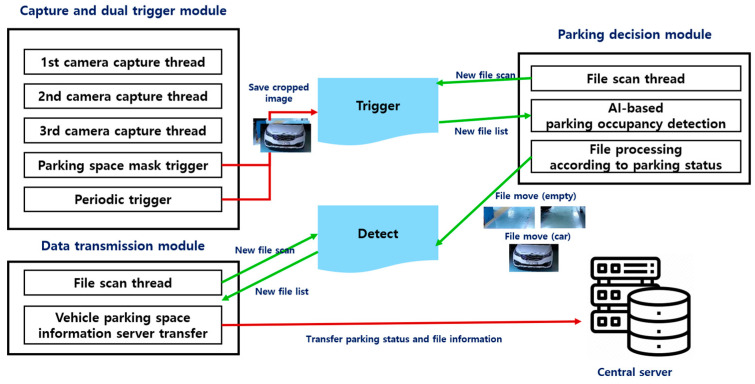
Overview of the system design and methodology.

The parking monitoring system proposed in this study is designed to provide real-time performance and high accuracy even in a limited resource environment. The proposed system is largely composed of four major modules: the IP camera frame capture thread, trigger algorithm, parking occupancy judgment module, and data transmission module. Each module is designed for efficient data processing, reduced computational load, and real-time information provision while being optimized to ensure reliability and stability even in an edge device environment. The proposed method is illustrated in Figure 2.

**Figure 3:**
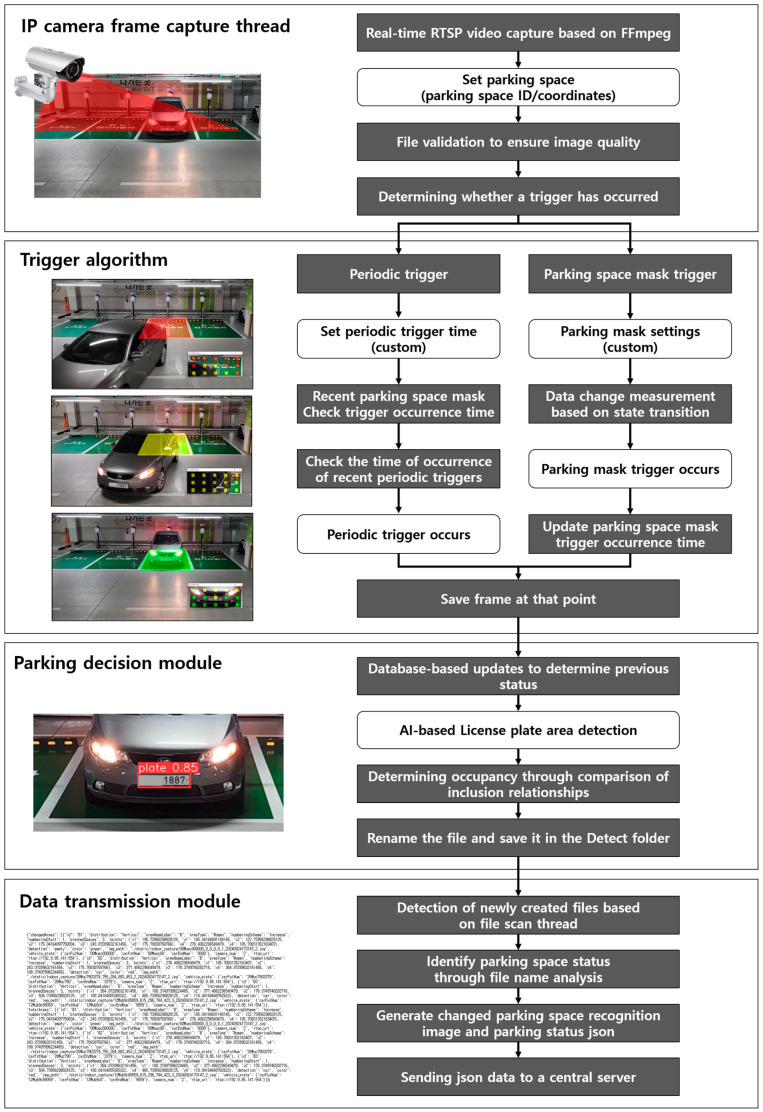
Scenario illustrating the proposed system.

The system operates under the scenario depicted in Figure 3. Its components are divided into four primary parts: the IP camera frame capture thread, trigger algorithm, parking decision module, and data transmission module. The first component, the IP camera frame capture thread, is essential for maintaining the system’s real-time capability and holds the base images used when subsequent computer vision algorithms detect changes in parking spaces. The second component involves a dual-trigger system, which includes a parking space mask trigger to efficiently utilize the limited computational power of IoT devices, and a periodic trigger to ensure the system’s reliability and consistency. When a change in the parking space is detected by the trigger algorithm, the image at that moment is cropped based on predefined parking space coordinates and saved in the trigger folder. The third component is the parking decision module. When the file scan thread detects a new file within the trigger folder, the system proceeds to utilize the AI-based algorithm sequentially to determine the presence of license plates and ascertain whether a vehicle occupies the parking space. Subsequently, the processed files are relocated to the detected folder with modified filenames in accordance with the recognition outcomes. The final component is the data transmission module, which transmits the detected results to the central server. It identifies the creation of a new file in the detect folder and a change in the parking space status. Subsequently, the system transmits the vehicle parking space information and file details to the central server.

### 3.1. IP Camera Frame Capture Thread

In this study, we designed an IP camera frame capture thread to stably and efficiently capture data even in resource-limited environments. This thread integrates various optimization techniques to efficiently utilize network bandwidth, reduce computational load, and ensure real-time performance. To collect real-time data from IP cameras, we used FFmpeg to capture frames via the RTSP protocol. FFmpeg is a lightweight, high-performance multimedia framework that guarantees the stability of RTSP streams and can effectively process real-time data. The default capture rate of FFmpeg is set to a fixed FPS. However, the proposed system automatically adjusts the capture rate according to the network status and resource usage by using a dynamic FPS adjustment algorithm.

(1) $$FPS=\min\;(\frac{R_{network}}{S_{frame}},\;{FPS}_{max}).$$

Here, R_network_ represents the current network bandwidth (bps); S_frame_ indicates the size of the frame (bytes); and FPS_max_ represents the maximum fps allowed by the camera.

Equation (1) yields the frame transmission limit according to the network bandwidth and determines the optimal FPS. On this basis, the FFmpeg command is dynamically updated to adjust the capture speed and is designed not to process more data than necessary. This minimizes the network load and computational amount, enabling efficient data processing even in a resource-limited environment.

The frame capture thread is designed asynchronously so that data capture and subsequent tasks are executed independently. By using Python 3.8’s threading library, capture and inference tasks are processed in separate threads, thereby preventing bottlenecks between tasks. This asynchronous processing structure contributed to maintaining real-time performance and maximizing system performance.

In addition, image validation was additionally implemented to increase the reliability of captured image data. The end marker of the image file was checked to determine whether data were corrupted, an error log was recorded in case of network connection problems or capture errors, and the camera was automatically restarted when a certain error occurrence threshold was exceeded to ensure stability.

The proposed IP camera frame capture thread enables stable and efficient data collection even in a resource-constrained environment and greatly improves the reliability and performance of the entire parking monitoring system. FFmpeg-based capture, dynamic FPS adjustment, asynchronous processing, and error recovery functions serve as an effective solution for real-time data processing in edge device environments.

### 3.2. Trigger Algorithm

The preprocessing methods implemented at the system edge comprise two types of triggers: a parking space mask and periodic trigger. The parking space mask trigger, designed for efficiently utilizing the system resources, is aimed at detecting the status of parking spaces in real-time and reducing unnecessary computations. The underlying concept of the parking space mask trigger is to monitor the change in parking space status and call the AI model only when a state transition such as parking or emptying occurs.

**Figure 4:**
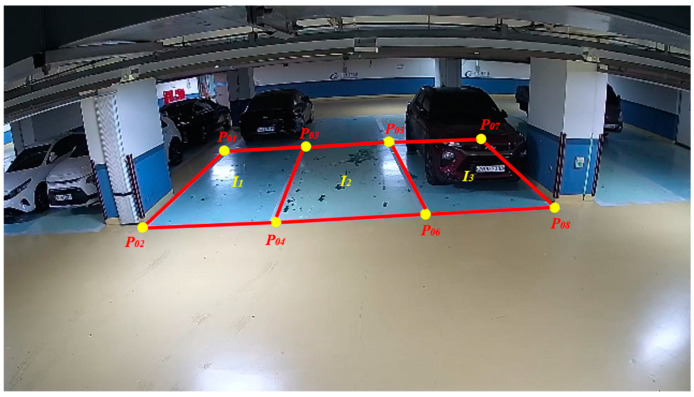
Defining the parking space area.

**Figure 5:**
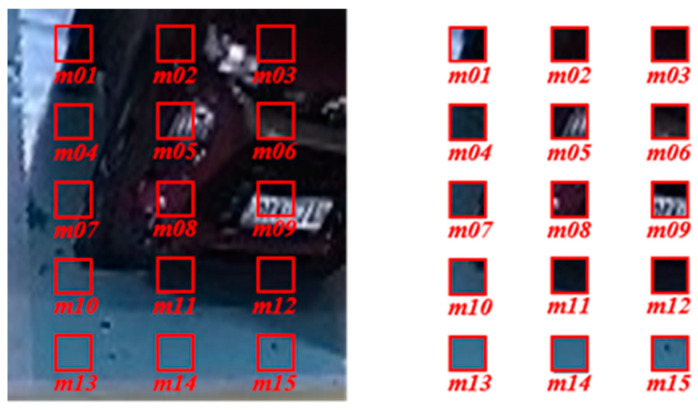
Parking space mask area definition.

The parking space mask trigger detects instances of vehicle parking or space departure by segmenting predefined parking space areas into mask units and monitoring the status change of each mask. The masks are generated based on user-defined rows and columns, and their sizes are determined by the image size and the number of masks set. As shown in Figure 4, the proposed method segments parking spaces (I_1_, I_2_, …, I_n_) based on predefined parking space area coordinates (P_1_, P_2_, …, P_m_). Each parking space I_n_ is defined by its boundary coordinates $I_{n}=\{P_{2n-1},P_{2n},P_{2n+1},P_{2n+2}\}$, which represent the vertices of a quadrilateral. To facilitate mask-based analysis, parking spaces are further divided into smaller masks (m_01_, m_02_, …, m_15_). The default configuration for vertical parking spaces consists of five rows and three columns, while for horizontal parking spaces, the configuration is three rows and five columns (Figure 5).

Once the parking space masks are configured, a mask state detection queue process runs to detect changes to each mask. As described in Equation (2), this process includes a pixel change detection process to ensure the accuracy and stability of the system.

(2) $$∆P_{m_{i,\;j}}=\frac{1}{N_{m_{i,\;j}}}\sum_{\left(x,y\right)\;\in \;m_{i,j}}\left|F_{t}\left(x,\;y\right)-F_{t-1}\left(x,y\right)\right|.$$

$∆P_{m_{i,\;j}}$: average pixel change in mask *j* of parking space *i*. This value serves as a critical metric for detecting state changes in the parking space.

$m_{i,j}$: a specific mask *j* within parking space *i*, which defines the localized region for detecting pixel changes.

$F_{t}(x,y)$: luminance value of the pixel (*x*, *y*) within the region of interest in the current frame *t*.

$F_{t-1}(x,y)$: luminance value of pixel (*x*, *y*) within the region of interest in the previous frame *t* − 1.

$N_{m_{i,j}}$: total number of pixels within the mask; $m_{i,j}$ represents the number of all pixels contained in a single mask area.

The workflow of Algorithm 1 shows a multi-step process for detecting changes in the status of a parking space. First, a mask is constructed based on the coordinates of the parking space, and the pixel changes in the mask are measured for each frame. In this process, the amount of pixel change is calculated using Equation (2), and, on this basis, a trigger is determined through a verification queue and a stabilization queue. The verification queue checks whether the pixel change exceeds the threshold, and the stabilization queue evaluates whether the change is consistent in consecutive frames. Finally, the stabilized results are synthesized to determine whether the final trigger is generated, and this value indicates whether the status of the parking space has changed. This method increases the trigger occurrence accuracy, ensures system reliability, and provides a solution suitable for real-time parking management.

**Figure 6:**
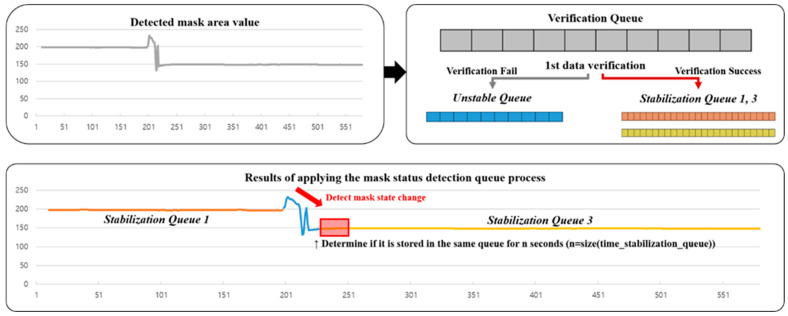
Real-world application of the single mask state detection queue process.

**Figure 7:**
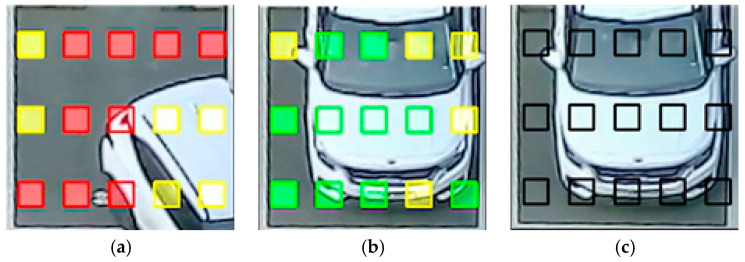
Intuitive implementation of the parking lot mask trigger process; (**a**) change occurrence and data validation; (**b**) trigger occurrence and data validation; and (**c**) determination completion.

Figure 6 illustrates the process of detecting a single mask state change through the step 1 and step 2 process of Algorithm 1 for the data collected during the parking process of an actual parked vehicle. The state of the parking space mask during the Parking Space Mask Trigger Process is visually represented in Figure 7. The change occurrence state is shown in red, which indicates that the data are unstable before detecting a continuous change, which may be a potential noise or a temporary change. The state during data verification is shown in yellow, which indicates that the current data are being filled in the stabilization queue. In the case of stable verification, it is shown in green, which indicates that the data are stably stored in the stabilization queue, the time-based verification is completed, and the trigger is generated. Finally, when the parking occupancy judgment module determines the state and completes processing through the final trigger decision process, it is shown in black so that the state after the trigger occurrence can be clearly distinguished visually.

**Algorithm 1:** Parking Space Mask Trigger Process**Require**: Streaming frame S, Parking space $I_{i}=\{P_{2i-1},P_{2i},P_{2i+1},P_{2i+2}\}$, User-defined thresholds ${T,T}_{m}$, Stabilization frames $n_{s}$ 1. **Input**: Streaming frame S 2. **Initial setup**: Configure parking lot masks $m_{i,j}$ based on $I_{n}$ 3. **Step 1**: Verification Queue 4. For each parking space ($I_{i}$): 5.        For each mask ($m_{i,j}$): 6.            $V_{m_{i,j}}$ = 1 if $∆P_{m_{i,j}}$ > T else $V_{m_{i,j}}$= 0 7. **Step 2**: Stabilization Queue 8. For each parking space ($I_{i}$): 9.        For each mask ($m_{i,j}$): 10.          $S_{m_{i,j}}$= 1 if $\sum_{k=1}^{n_{s}}V_{m_{i,j}}(t-k)=n_{s}$ else $S_{m_{i,j}}$= 0 11. **Step 3**: Final Trigger Decision 12. For each parking space ($I_{i}$): 13.      $T_{I_{i}}$ = 1 if $\sum_{j=1}^{n_{m}}S_{m_{i,j}}\geq \;T_{m}$ else $T_{I_{i}}$= 0 14. **Return** $T_{I_{i}}$

The parking space mask trigger approach minimizes unnecessary computations. However, relying solely on this approach can result in missing real changes, as the parking status is not updated in the absence of a trigger. To address this issue and ensure system resiliency, a periodic trigger is incorporated. The periodic trigger periodically re-evaluates the parking status, ensuring that the system periodically reviews the overall status even when there are no status changes.

Algorithm 2 describes a periodic trigger workflow that triggers status checks at fixed intervals, enabling the system to quickly respond to missing data or abnormal situations, thereby maintaining real-time detection capabilities. Combining the parking space mask-based trigger and the periodic trigger, the dual-trigger system addresses potential gaps in system responsiveness while enabling efficient and robust real-time parking space detection.
**Algorithm 2:** Periodic Trigger Process**Require**: Periodic trigger interval $T_{p}$ 1. **Initialize**: $T_{last\_trigger}\leftarrow 0$, $T_{periodic}\leftarrow 0$ 2. Repeat for every timestamp $T_{current}$: 3.       if $\left(T_{current}-T_{last\_trigger}\right)\geq T_{p}$: 4.          $T_{periodic}\;\leftarrow 1$                 ▷ Set trigger decision to active 5.          $t_{last\_trigger}\leftarrow t_{current}$           ▷ Update the last trigger timestamp 6.       else: 7.          $T_{periodic}\leftarrow 0$ 8. **Return** $T_{periodic}$

### 3.3. Parking Decision Module

This module is designed to efficiently determine the occupancy status of a parking space by utilizing a predefined parking area $I_{n}$ and AI-based license plate detection. The status of a parking space is determined by comparing the license plate area detected in the image frame at the time of trigger occurrence with the predefined parking space coordinates. This allows for the simultaneous evaluation of multiple parking space states while minimizing the number of AI model executions, maintaining high accuracy and computational efficiency.

Algorithm 3 can be used to determine the occupancy state based on the relationship between the license plate bounding box $B_{j}$, parking space $I_{n}$, and additional conditions $T_{A}\;and\;T_{R}$. The workflow of the parking decision process begins with the use of the AI model to detect all bounding boxes $B_{j}$ in the triggered image S. For each parking space, the algorithm evaluates whether the detected bounding boxes are completely contained within the parking space $I_{n}$. Additionally, the bounding boxes are validated based on their area and aspect ratio thresholds, removing small boxes caused by double parking or noise and filtering out vertically elongated boxes to prevent FP values. This approach ensures high accuracy and computational efficiency, maintaining robust condition detection while minimizing unnecessary computations.
**Algorithm 3:** Parking Decision Process**Require**: Triggered image S, Parking spaces $I_{n}$, Thresholds $T_{A}\;and\;T_{R}$ 1. **Input**: Triggered image S, Parking spaces $I_{n}$ 2. **Detection**: Use the AI model to detect all bounding boxes $B_{j}$ in S 3. For each parking space ($I_{n}$): 4.        For each bounding box ($B_{j}$): 5.           if $B_{j}\subseteq \;I_{n}\;and\;A\left(B_{j}\right)\geq T_{A}\;and\;R(B_{j})\leq T_{R}$: 6.              $P_{state}\left(I_{n}\right)\leftarrow “Car”$ 7.           else: 8.              $P_{state}\left(I_{n}\right)\leftarrow “Empty”$ 9. **Return**
$P_{state}\left(I_{n}\right)$

## 4. Experimental Setting

The edge device used in this experiment was Odroid N2+ (Hardkernel Co., Ltd., Anyang, Gyeonggi Province, Republic of Korea), which was selected to evaluate real-time computation and data processing capabilities. The main specifications of Odroid N2+ are summarized in Table 2, and this device is designed to maximize system efficiency and operate stably even in limited resource environments.

This experiment was conducted in the parking facility of the College of Semiconductor, Gachon University. Before conducting field tests, a dataset of videos and images capturing parking and exits was collected over more than two weeks. We used it to configure the system parameters for the field test and train the AI model.

**Figure 8:**
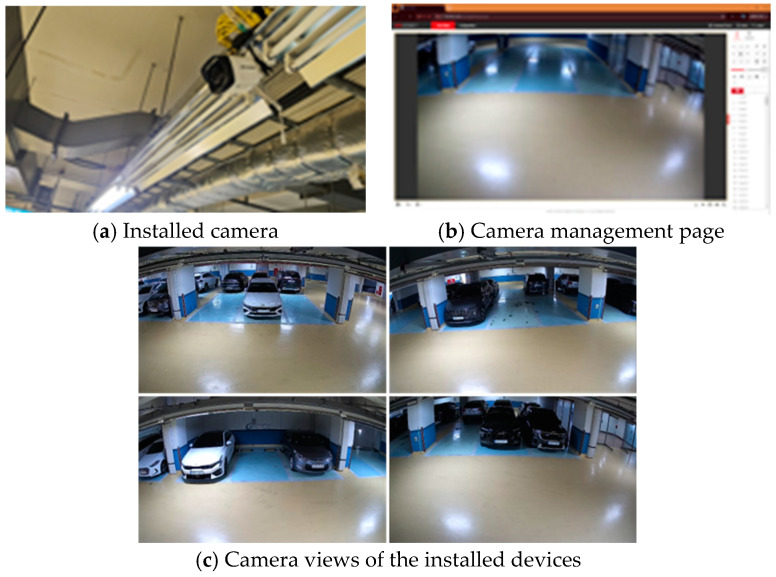
Building a field test environment.

The field experiment was conducted on a total of 12 parking spaces in the 2nd basement floor parking lot, and four DS-2CD1021-I IP cameras (HiKVISION Co., Ltd., Guangzhou, China) were installed to monitor them. All the cameras were placed in locations where they could clearly observe the parking spaces and were used to evaluate the performance of the parking space detection and trigger generation algorithm. The experimental site is visually represented in Figure 8, including the camera installation scene, camera management interface, and field of view for monitoring the actual parking spaces.

## 5. Results and Discussion

This section evaluates the performance of the proposed parking monitoring system across three critical areas: model performance on the edge device, trigger algorithm efficiency, and real-world field tests using the dual-trigger mechanism with the SSD-MobileNetv2 model. The experiments were designed to assess accuracy, efficiency, and reliability under various conditions to ensure the system’s applicability in real-time parking management scenarios. Performance metrics were calculated using true-positive (TP), true-negative (TN), false-positive (FP), and false-negative (FN) values, from which accuracy, precision, recall, and F1-score were derived using Equations (3)–(6).

(3) $$Accuacy=\frac{TP+TN}{TP+TN+FP+FN}$$

(4) $$Precision=\frac{TP}{TP+FP}$$

(5) $$Recall=\frac{TP}{TP+FN}$$

(6) $$F1\text{-}\mathrm{score}=2\cdot \frac{precision\cdot recall}{precision+recall}.$$

### 5.1. Edge Device-Based AI Model Performance Evaluation

To evaluate the suitability of AI models for real-time parking monitoring, three models were tested: YOLOv8n, YOLOv8n (converted to ONNX with FP16 optimization), and SSD-MobileNet v2. The evaluation focused on inference time and performance metrics, including TP, FP, FN, precision, recall, and F1-score. These metrics are essential to determine the balance between accuracy and computational efficiency, which are critical in resource-constrained environments such as parking management systems. The models were tested on a dataset of 20,000 images, reflecting various parking scenarios captured during system development. The results are summarized in Table 3.

The results show that YOLOv8n achieved the highest precision and recall, demonstrating its superior accuracy in detecting parking status changes. However, its inference time exceeded real-time requirements, making it unsuitable for the dynamic environment of a parking management system. YOLOv8n (ONNX + FP16) slightly improved computational efficiency by reducing model size and floating-point operations. However, its inference time increased compared to the original YOLOv8n. This result is attributed to the additional overhead in handling the ONNX format and FP16 precision optimization, combined with the fact that the edge device used in this study relies solely on the CPU for inference. Without dedicated GPU or specialized hardware support for FP16 operations, the potential benefits of optimization could not be fully realized.

In contrast, SSD-MobileNet v2 demonstrated the fastest inference time, meeting real-time requirements while maintaining competitive performance metrics. Its precision, recall, and F1-score indicate sufficient reliability for field tests, ensuring that the system can detect parking changes accurately within the constraints of limited resources. Given these results, SSD-MobileNet v2 was selected for deployment in the field test to balance real-time processing needs and system reliability effectively.

### 5.2. Trigger Algorithm Performance Evaluation

The trigger algorithm was evaluated by comparing three configurations: the periodic trigger, parking space mask trigger, and dual trigger. The periodic trigger in the dual-trigger configuration was set to a longer interval (5 min) compared to the standard periodic trigger (5 s), ensuring system reliability while maintaining efficiency. Experiments were conducted under two scenarios: a relatively idle period (weekend, 48 h) and a busy period (weekday, 48 h). The evaluation measured the total number of verification cycles and the accuracy of detecting actual parking and departure events. The results are summarized in Table 4.

One key consideration in the evaluation is the exclusion of true-negatives (TNs). As periodic triggers generate verification cycles regardless of parking space status, a TN is not meaningful in this context. For example, periodic triggers are designed to ensure updates at regular intervals, even when no parking or departure events occur. As a result, the high frequency of periodic triggers makes the TN metric irrelevant for assessing system performance. Instead, metrics such as TP, FP, and FN better indicate the system’s effectiveness in detecting state changes.

The recall metric was selected as the primary measure of accuracy to emphasize the system’s ability to detect actual parking and departure events. Recall is particularly important in this application because missing a state change (i.e., FN) can undermine the reliability of the parking management system. By prioritizing recall, the evaluation highlights the system’s effectiveness in ensuring that no valid parking or departure event is missed.

The parking space mask trigger demonstrated resource efficiency by minimizing unnecessary computations. However, under unstable network conditions, it occasionally failed to generate triggers, resulting in FN values. To mitigate this limitation, the dual-trigger mechanism was introduced, combining the computational efficiency of the parking space mask trigger with the reliability of the periodic trigger.

The dual-trigger approach performed well across all scenarios, leveraging periodic reinforcements at 5 min intervals to address potential network instability. This configuration provides a balanced performance, with higher recall and optimized resource usage, making it a robust solution for real-time parking management.

### 5.3. Field Validation with Dual Trigger and SSD-MobileNetv2

**Figure 9:**
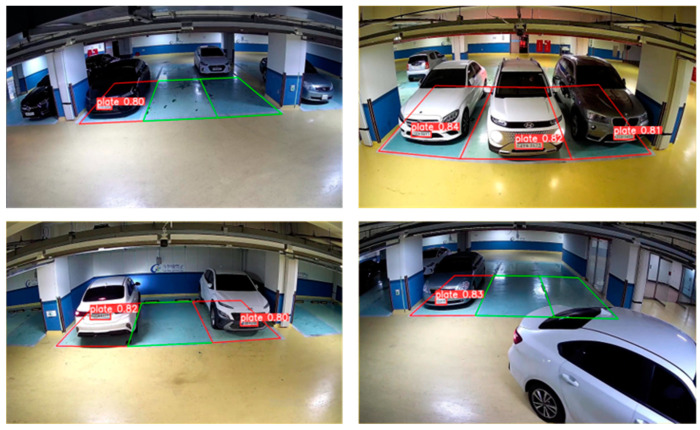
Sample detection results with our proposed system.

The field validation was conducted to evaluate the practical applicability of the dual-trigger mechanism combined with the SSD-MobileNet v2 model in a real-world parking management system. The experiment spanned four months and monitored 12 parking spaces using four IP cameras installed in the parking facility of Gachon University’s Semiconductor College. The dual-trigger mechanism, integrating periodic and parking space mask triggers, was designed to enhance detection reliability while minimizing computational overhead. Figure 9 illustrates the sample detection results.

During the validation period, the system continuously processed real-time image data to detect parking and departure events. The SSD-MobileNet v2 model was selected for its ability to meet real-time requirements with competitive accuracy, as demonstrated in the previous evaluation.

The results demonstrate that the system maintained a high level of accuracy (99.58%) and recall (99.95%), indicating its capability to detect almost all parking and departure events. The precision (96.99%) and F1-score (98.48%) further confirm the system’s robustness in handling real-world scenarios, including occasional anomalies such as lighting changes, partial occlusions, and network instabilities.

The integration of the dual-trigger mechanism proved effective in balancing reliability and computational efficiency. The periodic trigger addressed scenarios where network instabilities or environmental factors might cause the parking space mask trigger to miss events. By ensuring periodic system checks, the dual-trigger mechanism provided a safety net, enhancing overall system dependability.

These findings validate the proposed system’s feasibility for real-world deployment, highlighting its ability to manage parking spaces efficiently and reliably while adhering to real-time constraints. The combination of SSD-MobileNet v2 and the dual-trigger mechanism demonstrates significant potential for broader adoption in similar parking management scenarios.

### 5.4. Comparison with Other Research

Table 5 summarizes the existing parking monitoring systems and the system proposed in this study. Ling et al. [36] built a system based on IoT devices using a single video input and adopted a classification method that employed Haar and an F-test as the primary algorithms. However, their study faced limitations in terms of a lack of training datasets and insufficient image processing speed and system accuracy, preventing it from being considered a real-time parking monitoring system. This study leverages SSD-MobileNet, which processes a single image in approximately 0.32 s, thereby enabling real-time parking monitoring. By processing data locally on IoT devices and only transmitting the parking occupancy results to the server, this system minimizes network traffic and latency, distinguishing itself from previous research. Nieto et al. [37] developed a system capable of processing multiple IP camera streams in a desktop environment using faster R-CNN and fusion techniques. While their study shares similarities with the current research in terms of handling multiple video inputs, it does not utilize IoT devices, thereby differing in terms of resource efficiency. The system proposed in this study has been designed to process up to three simultaneous IP camera streams on edge devices.

Ke et al. [24] employed the SSD and SORT algorithms to detect objects in indoor and outdoor parking lots. They then proceeded to implement a system that integrated IoT devices and servers. However, their study did not consider the efficiency of the resources used for real-time occupancy detection nor did it address the periodic operation of the algorithm. By contrast, this study introduces a trigger-based algorithm that executes occupancy detection only when there is a change in the state of the parking spaces, thereby reducing unnecessary computations. Consequently, this results in the efficient utilization and optimization of system resources. Nguyen et al. [38] constructed an IoT-device-based system utilizing mAlexNet for object classification. This system employs a periodic algorithmic approach and was validated over a three-day period across 24 parking lots. However, the periodic execution method can result in unnecessary computations and inefficiencies in resource usage. By contrast, this study utilizes a trigger-based algorithm and executes occupancy detection only upon state changes in the parking spaces, thereby minimizing unnecessary computations. This approach enables the efficient management of resources and reduces energy consumption in IoT environments.

In conclusion, while previous research has primarily focused on object detection or classification algorithms, this study aimed to optimize resource management and real-time processing. The system was designed to be customizable to the installation environment and the devices that are in use. Following a four-month deployment and validation period in real-world conditions, the system achieved an accuracy rate of 98.48%, surpassing the performance of existing studies and demonstrating its stability and reliability across real-world scenarios. Furthermore, the system can process a single image in approximately 0.32 s, demonstrating faster processing speeds compared with those of the existing system.

## 6. Conclusions

This study proposed a resource-efficient real-time parking management system integrating a dual-trigger mechanism and the SSD-MobileNet v2 model. The system effectively addresses computational limitations and enhances detection reliability for real-time parking status updates.

The experimental results validate the system’s effectiveness, particularly the dual-trigger mechanism, which combines periodic and parking space mask triggers. This approach demonstrated improved accuracy and robustness, resolving issues such as network instability while optimizing computational efficiency. Field validation over a four-month period confirmed the system’s practical applicability, achieving a recall of 99.95%, an accuracy of 99.58%, and an F1-score of 0.9848. These results highlight the system’s capability to reliably detect parking status changes with minimal computational overhead.

However, some areas for further research remain. To further enhance the system, future research will focus on evaluating its performance in outdoor parking lots, where external factors such as weather conditions and lighting changes can affect detection accuracy. In addition, the system will be tested alongside other parking detection systems under identical conditions to provide a more comprehensive performance comparison.

Another key area of improvement is enhancing system scalability to accommodate larger parking facilities. As the number of parking spaces increases, expanding the system efficiently requires careful consideration of both hardware performance and camera placement strategies. To address this, two potential solutions are being explored:

1. Enhancing Computational Efficiency: As the number of cameras and data volume increase, ensuring real-time processing becomes crucial. This can be achieved through hardware performance enhancement, such as utilizing more powerful AI accelerators or deploying multiple edge devices for distributed processing. Additionally, model optimization techniques, including ONNX conversion and FP16 quantization, can reduce computational load while maintaining high detection accuracy.
2. Vehicle-Based Detection Instead of License Plate Recognition: The current system relies on license plate detection, which requires cameras to be positioned at angles where plates are visible. However, an alternative approach is vehicle-based detection using a top-down perspective, allowing a single camera to monitor multiple parking spaces simultaneously. This method eliminates the dependency on license plate visibility, making the system more scalable and flexible. This approach is currently being explored in ongoing research on outdoor parking monitoring, utilizing top-view cameras to detect vehicles based on their position and movement patterns.

Furthermore, a critical aspect for future work is the detailed analysis of computational overhead and system-wide latency. While this study optimized AI model execution through the dual-trigger mechanism and selected SSD-MobileNet v2 for a balance between accuracy and speed, a comprehensive performance evaluation of processing latency at each stage—including image acquisition, trigger processing, AI inference, and data transmission—will be conducted in follow-up research. Additionally, optimization techniques such as model quantization, hardware acceleration (e.g., TensorRT and OpenVINO), and multi-threaded processing will be explored to further enhance real-time performance.

By addressing these aspects in future work, the system can be refined to achieve higher scalability, lower latency, and increased computational efficiency, making it a more viable solution for large-scale deployment in diverse parking environments, including outdoor settings with varying lighting and weather conditions.

Additionally, improving system accessibility through the development of a web-based user interface is another important direction. This enhancement would provide end users with intuitive monitoring and control capabilities, further broadening the system’s usability. Additionally, creating a centralized management system that integrates and manages multiple IoT devices would enable large-scale smart parking management across multiple facilities. This would pave the way for the seamless implementation of smart city infrastructure.

In conclusion, the proposed system demonstrates high efficiency and reliability, offering a robust solution for real-time parking management in resource-constrained environments. By addressing existing limitations and expanding its capabilities through further research, the system can serve as a cornerstone for future advancements in intelligent parking solutions and smart city applications.

## Figures and Tables

**Table 2:** IoT device specifications.

Item	Specifications
CPU	Quad-core Cortex-A73/Dual-core Cortex-A53
RAM	4 GB LPDDR4-3200 SDRAM
GPU	Mali-G52 GPU
Storage	SD Card 32 GB
OS	Debian-installer-11-netboot-amd64 (20210731 + deb11u10)
Size	90 mm × 90 mm × 17 mm
Weight	Approximately 132 g

**Table 3:** Performance comparison of edge device-based AI models.

Model	Inference Time	Accuracy (%)	Precision (%)	Recall (%)	F1-Score
YOLOv8n	1.58 s per frame	99.97%	100%	99.94%	0.9997
YOLOv8n (ONNX + FP16)	1.88 s per frame	99.95%	99.95%	99.94%	0.9994
SSD-MobileNet v2	0.32 s per frame	99.65%	99.73%	99.72%	0.9972

**Table 4:** Performance comparison of trigger algorithms.

Trigger Type	Scenario	Total Trigger	TP	FP	FN	Recall (%)	CPU Usage (%)	Memory Usage (%)
Periodic trigger	Weekend	34,560	10	34,550	0	100%	69.21%	36.10%
	Weekday	34,560	210	34,350	0	100%	71.13%	39.44%
Parking space Mask trigger	Weekend	32	10	22	0	100%	42.98%	56.20%
	Weekday	548	207	338	3	98.57%	43.62%	56.78%
Dual trigger	Weekend	608	10	598	0	100%	55.16%	55.93%
	Weekday	1124	210	914	0	100%	59.05%	56.57%

**Table 5:** Comparison of the proposed system with existing parking monitoring systems.

Research Work	Ling et al. 2017 [36]	Nieto et al. 2019 [37]	Ke et al. 2020 [24]	Nguyen et al. 2021 [38]	This Study
System input	Single video	Multiple videos (IP camera)	Single video	Single video	Multiple videos (IP camera)
Development environment	IoT devices	Desktop	IoT devices	IoT devices	IoT devices
Pipeline logic	Classification	Detection	Detection	Classification	Detection
Applied algorithms	Haar and F-test	Faster R-CNN and fusion	SSD, BG, SORT, and fusion	mAlexNet	Trigger algorithm and SSD-MobileNet v2
Algorithm operation cycle	NA	NA	NA	Every 30 s	When trigger occurs
Training data	469 frames	6616 frames	127,125 frames	11,760 patches	137,550 frames
Validation data	90 detections	2200 images	3-month real-word validation and 10 parking lots (outdoor) + 6 parking lots (indoor)	Three-day real-world validation and 24 parking lots (outdoor)	4-month real-world validation and 12 parking lots (indoor)
Test environment	Outdoor	Outdoor	Outdoor and indoor	Outdoor	Indoor
Image processing speed	1 frame per 5 s	NA	1 frame per 1 s	1 frame per 0.743 s	1 frame per 0.32 s
System accuracy	91%	>91%	95.6%	>97%	98.48%

## Data Availability

Data are contained within the article.

## References

[1] Hedges & Company How Many Cars Are There in the World? Hedges & Company Blog. https://www.whichcar.com.au/news/how-many-cars-are-there-in-the-world.

[2] Towne Park Parking Statistics. Towne Park. https://www.townepark.com/parking-statistics/.

[3] Cao K., Liu Y., Meng G., Sun Q. (2020). An overview on edge computing research. IEEE Access.

[4] Shi W., Cao J., Zhang Q., Li Y., Xu L. (2016). Edge computing: Vision and challenges. IEEE Internet Things J..

[5] Yang P.A., Hu X., Li R., Zhou Z., Gui Y., Sun R., Wu D., Wang X., Bian X. (2025). Flexible magnetoelectric sensors with enhanced output performance and response time for parking spaces detection systems. Sens. Actuators A Phys..

[6] Allbadi Y., Shehab J.N., Jasim M.M. (2021). The smart parking system using ultrasonic control sensors. IOP Conference Series: Materials Science and Engineering.

[7] Al-Turjman F., Malekloo A. (2019). Smart parking in IoT-enabled cities: A survey. Sustain. Cities Soc..

[8] Lin T., Rivano H., Le Mouël F. (2017). A survey of smart parking solutions. IEEE Trans. Intell. Transp. Syst..

[9] Zhang Z., Li X., Yuan H., Yu F. (2013). A street parking system using wireless sensor networks. Int. J. Distrib. Sensor Netw..

[10] Ratti S.A., Pirzada N., Shah S.M.A., Naveed A. (2023). Intelligent Car Parking System Using WSN. Proceedings of the 2023 Global Conference on Wireless and Optical Technologies (GCWOT).

[11] Zhang Z., Tao M., Yuan H. (2015). A parking occupancy detection algorithm based on AMR sensor. IEEE Sens. J..

[12] Jeon Y., Ju H.-I., Yoon S. Design of an LPWAN communication module based on secure element for smart parking application. Proceedings of the IEEE International Conference on Consumer Electronics (ICCE).

[13] Lou L., Zhang J., Xiong Y., Jin Y. (2019). An improved roadside parking space occupancy detection method based on magnetic sensors and wireless signal strength. Sensors.

[14] Yamada S., Watanabe Y., Kanamori R., Sato K., Takada H. (2022). Estimation method of parking space conditions using multiple 3D-LiDARs. Int. J. ITS Res..

[15] De Almeida P.R., Oliveira L.S., Britto A.S., Silva E.J., Koerich A.L. (2015). PKLot–A robust dataset for parking lot classification. Expert Syst. Appl..

[16] Baroffio L., Bondi L., Cesana M., Redondi A.E., Tagliasacchi M. A visual sensor network for parking lot occupancy detection in smart cities. Proceedings of the IEEE 2nd World Forum Internet Things (WF-IoT).

[17] Bulan O., Loce R.P., Wu W., Wang Y., Bernal E.A., Fan Z. (2013). Video-based real-time on-street parking occupancy detection system. J. Electron. Imaging.

[18] Amato G., Carrara F., Falchi F., Gennaro C. Vairo Car parking occupancy detection using smart camera networks and deep learning. Proceedings of the 2016 IEEE Symposium on Computers and Communication.

[19] AFarley, Ham H. (2021). Real time IP camera parking occupancy detection using deep learning. Procedia Comput. Sci..

[20] Acharya D., Yan W., Khoshelham K. (2018). Real-time image-based parking occupancy detection using deep learning. Res. Locate.

[21] Xie Z., Wei X. Automatic parking space detection system based on improved YOLO algorithm. Proceedings of the 2021 2nd International Conference on Computer Science and Management Technology (ICCSMT).

[22] Carrasco D.P., Rashwan H.A., García M.Á., Puig D. (2023). T-YOLO: Tiny Vehicle Detection Based on YOLO and Multi-Scale Convolutional Neural Networks. IEEE Access.

[23] Vítek S., Melničuk P. (2017). A distributed wireless camera system for the management of parking spaces. Sensors.

[24] Ke R., Zhuang Y., Pu Z., Wang Y. (2020). A smart, efficient, and reliable parking surveillance system with edge artificial intelligence on IoT devices. IEEE Trans. Intell. Transp. Syst..

[25] Falaschetti L., Manoni L., Palma L., Pierleoni P., Turchetti C. (2024). Embedded Real-Time Vehicle and Pedestrian Detection Using a Compressed Tiny YOLO v3 Architecture. IEEE Trans. Intell. Transp. Syst..

[26] Ming P.Y.K., Khan N.A., Asirvatham D.A., Tayyab M., Balakrishnan S.A., Kumar D. Detecting Street Parking Occupancy Using Image Recognition with Yolo. Proceedings of the 2024 International Conference on Emerging Trends in Networks and Computer Communications (ETNCC).

[27] Siddiqui S.Y., Khan M.A., Abbas S., Khan F. (2022). Smart occupancy detection for road traffic parking using deep extreme learning machine. J. King Saud Univ.-Comp. Inf. Sci..

[28] Paidi V., Fleyeh H., Nyberg R.G. (2020). Deep learning-based vehicle occupancy detection in an open parking lot using thermal camera. IET Intell. Transp. Syst..

[29] Girshick R., Donahue J., Darrell T., Malik J. Rich feature hierarchies for accurate object detection and semantic segmentation. Proceedings of the IEEE Conference on Computer Vision and Pattern Recognition (CVPR).

[30] Ren S. (2015). Faster R-CNN: Towards real-time object detection with region proposal networks. arXiv.

[31] Redmon J., Divvala S., Girshick R., Farhadi A. You only look once: Unified real-time object detection. Proceedings of the IEEE Conference on Computer Vision and Pattern Recognition.

[32] Liu W., Anguelov D., Erhan D., Szegedy C., Reed S., Fu C.-Y., Berg A.C. SSD: Single shot multibox detector. Proceedings of the European Conference Computer Vision.

[33] Alqahtani D.K., Cheema M.A., Toosi A.N. (2024). Benchmarking deep learning models for object detection on edge computing devices. Proceedings of the International Conference on Service-Oriented Computing.

[34] Tensorflow Hub. https://www.kaggle.com/models/tensorflow/ssd-mobilenet-v2/tensorFlow2/ssd-mobilenet-v2.

[35] Park S.W., Park Y.J., Choi H.W., Ha S.H., Do Y.S. (2024). Real-time Object Detection Model for Raspberry Pi. Proceedings of the Annual Conference of KIPS.

[36] Ling X., Sheng J., Baiocchi O., Liu X., Tolentino M.E. Identifying parking spaces & detecting occupancy using vision-based IoT devices. Proceedings of the Global Internet Things Summit (GIoTS).

[37] Nieto R.M., García-Martín Á., Hauptmann A.G., Martínez J.M. (2019). Automatic vacant parking places management system using multicamera vehicle detection. IEEE Trans. Intell. Transp. Syst..

[38] Nguyen T., Tran T., Mai T., Le H., Le C., Pham D., Phung K.H. An adaptive vision-based outdoor car parking lot monitoring system. Proceedings of the 2020 IEEE Eighth International Conference on Communications and Electronics (ICCE).

